# Trypanosome diversity in small mammals in Uganda and the spread of *Trypanosoma lewisi* to native species

**DOI:** 10.1007/s00436-023-08048-2

**Published:** 2023-12-16

**Authors:** Waswa Sadic Babyesiza, Abdul Katakweba, Alena Fornůsková, James Ssuunaf, Sisiria Akoth, Joseph Mpagi, Joelle Goüy de Bellocq, Josef Bryja, Jan Votýpka

**Affiliations:** 1 Institute of Vertebrate Biology of the Czech Academy of Sciences, Květná 8, 603 65 Brno, Czech Republic; 2 Africa Centre of Excellence for Innovative Rodent Pest Management and Biosensor Technology Development (ACE IRPM&BTD, Institute of Pest Management Centre, Sokoine University of Agriculture, Morogoro, Tanzania; 3 Department of Wildlife Management, Sokoine University of Agriculture, Morogoro, Tanzania; 4 Department of Zoology, Entomology and Fisheries Science, Makerere University, Kampala, Uganda; 5 Department of Microbiology and Immunology, Busitema University, Mbale, Uganda; 6 Institute of Parasitology, Biology Centre, Czech Academy of Sciences, České Budějovice, Czech Republic; 7 Department of Parasitology, Faculty of Science, Charles University, Prague, Czech Republic

**Keywords:** Muridae, Soricidae, *Herpetosoma*, *Aneza*, *Ornithotrypanum*

## Abstract

Uganda’s diverse small mammalian fauna thrives due to its rich habitat diversity, which hosts a wide range of blood parasites, including trypanosomes, particularly the subgenus *Herpetosoma* typical for rodent hosts. We screened a total of 711 small mammals from various habitats for trypanosomes, with 253 microscopically examined blood smears and 458 tissue samples tested by nested PCR of the 18S rRNA gene. Of 51 rodent and 12 shrew species tested, microscopic screening reaches 7% overall prevalence (with four rodent species positive out of 15 and none of the shrew species out of four), while nested PCR indicated a prevalence of 13% (17 rodent and five shrew species positive out of 49 and 10, respectively). We identified 27 genotypes representing 11 trypanosome species, of which the majority (24 genotypes/9 species) belong to the *Herpetosoma* subgenus. Among these, we detected 15 new genotypes and two putative new species, labeled AF24 (found in *Lophuromys woosnami*) and AF25 (in *Graphiurus murinus*). Our finding of three new genotypes of the previously detected species AF01 belonging to the subgenus *Ornithotrypanum* in two *Grammomys* species and *Oenomys hypoxanthus* clearly indicates the consistent occurrence of this avian trypanosome in African small mammals. Additionally, in *Aethomys hindei*, we detected the putative new species of the subgenus *Aneza*. Within the *T. lewisi* subclade, we detected eleven genotypes, including six new; however, only the genotype AF05b from *Mus* and *Rattus* represents the invasive *T. lewisi*. Our study has improved our understanding of trypanosome diversity in African small mammals. The detection of *T. lewisi* in native small mammals expands the range of host species and highlighting the need for a broader approach to the epidemiology of *T. lewisi.*

**Supplementary Information:**

The online version contains supplementary material available at 10.1007/s00436-023-08048-2.

## Introduction

Small mammals, represented by rodents and shrews in this study, are one of the most abundant mammals on Earth. Since numerous live in synanthropic habitats, they are reservoirs of several pathogens transmissible to humans (Meerburg et al. 2009). Within blood parasites, rodents are important hosts of trypanosomes (e.g., Schwan et al. 2016). Trypanosomes are often transmitted by a bite of hematophagous vectors; however, rodents and insectivores can become infected by ingestion of infected insects like fleas (e.g., Dahesh and Mikhail 2016).

The number of known trypanosome species (genus *Trypanosoma*) is steadily growing, including in Africa (e.g., Adams et al. 2010; Votýpka et al. 2022), and the expanding diversity is accommodated into 16 subgenera (Kostygov et al. 2021). Host specificity varies widely, from extremely low in the subgenera *Squamatrypanum* and *Haematomonas* to the highly host-specific subgenus *Herpetosoma*, whose species are usually restricted to a single host species/genus of rodents and/or insectivores (Noyes et al. 2002; Kostygov et al. 2021; Votýpka et al. 2022). Only *Trypanosoma* (*Herpetosoma*) *lewisi* stands out in this regard. Originally hosted by commensal Rattini rodents (*Rattus rattus*, *R. exulans*, and *R. norvegicus*), it has been spread beyond its original range due to human migration and trade. As a result, it has been found in more than a hundred rodent species worldwide, including Africa. Additionally, nonrodent species, such as shrews (Pumhom et al. 2014), marsupials (Pinto et al. 2006), bats (Fox and Thillet 1962), and primates (Maia da Silva et al. 2010), have also been reported as hosts. Even though *T. lewisi* is largely nonpathogenic in most rodent species, it is argued to have been at the center of the extinction of the native Maclear’s Rat (*Rattus macleari*) on Christmas Island (Wyatt et al. 2008). *T. lewisi* is recognized as a zoonotic species, and human infections, including fatal cases, have been reported from Asia and Africa (Truc et al. 2013; Lun et al. 2015).

In contrast to morphology-based identification, lacking reliable diagnostic features (Hoare 1972; Kaufer et al. 2017) and underestimating prevalence and diversity (Schwan et al. 2016; Ortiz et al. 2018), recent developments in molecular identification techniques and phylogenetic analysis (Adams et al. 2010; Votýpka et al. 2022) have improved understanding and appreciation of trypanosome diversity. The subgenus *Herpetosoma* has about 50 nominal species that were named based on their morphology and putative confinement to the host species (Noyes et al. 2002; Dybing et al. 2016). The current determination of trypanosomes in vertebrate hosts or invertebrate vectors is based on conserved gene sequences, such as the 18S rRNA and glyceraldehyde 3-phosphate dehydrogenase genes (Hamilton et al. 2007; Pumhom et al. 2014; Gibson 2017; Hamilton and Stevens 2017; Kostygov et al. 2021), and leads to a significant increase in the current trypanosome diversity, including that in rodents (Sato et al. 2007; Maia da Silva et al. 2010; Votýpka et al. 2022).

Although trypanosome infections of small mammals remain significant public health concerns for humans and animals worldwide, there is a lack of comprehensive research on trypanosome infections in wildlife reservoir hosts, particularly in Africa (Kasozi et al. 2021). Various *Trypanosoma* species associated with small mammals have been identified across Africa (Dobigny et al. 2011; Schwan et al. 2016; Tatard et al. 2017; Votýpka et al. 2022), including Uganda (Salzer et al. 2016). Although several studies have explored the occurrence of trypanosomes in different habitats, in Uganda, no investigation has been carried out specifically examining the prevalence and diversity of trypanosomes in small mammals across various habitats, employing molecular analysis to uncover their true diversity.

## Material and methods

### Study area

**Fig. 1:**
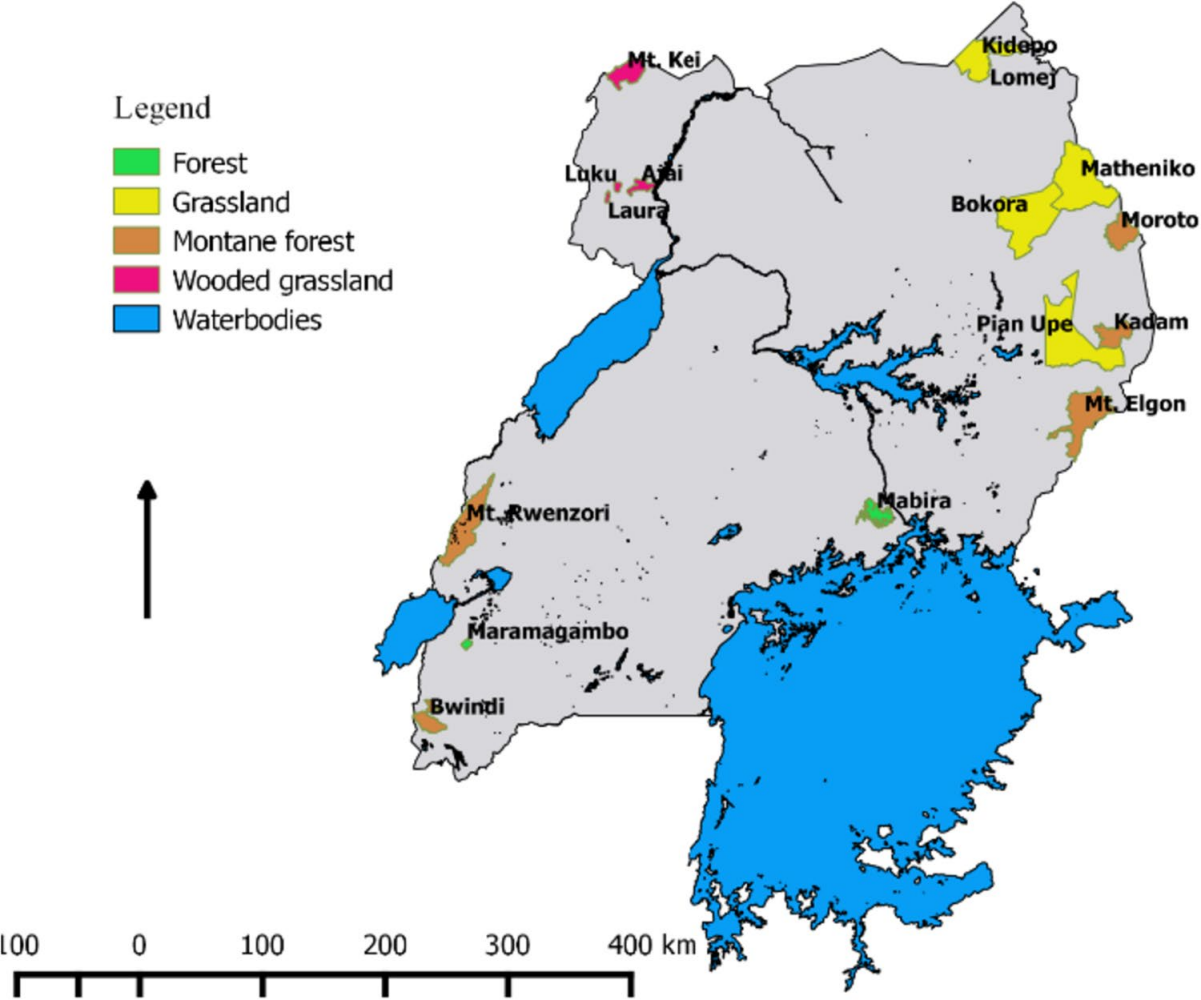
View into the study areas showing the main vegetation types in each survey location; note that bushed fallows are not indicated because they bordered most vegetation types as an ecotone between protected area and communities

The study was conducted between 2018 and 2022 across various habitats in Uganda, encompassing grasslands, bushed fallows, woodlands, montane forests, and lowland forests in Karamoja, West Nile, and the Albertine Rift (Fig. 1). In Karamoja, the surveyed sites included Kidepo Valley National Park, known for its grasslands and wooded grasslands, as well as the Matheniko-Bokora Wildlife Reserve, characterized by grasslands. Mount Moroto exhibited degraded woodlands on its slopes, pristine forests, *Combretum* woodlands, and bushland/tree/shrub-steppe at its peak. Mount Kadam featured secondary regenerating montane forests bordering grassland plains and acacia savannas in the Pian Upe reserve. On the slopes of Mount Elgon, the survey covered areas adjacent to forests and fallow agricultural fields in local communities representing bushed fallows. In West Nile, the study sites included woodland in forest reserves such as Mount Kei, Luku, Laura, and Ajai, along with riverine forests in Ajai. Maramagambo Forest in Queen Elizabeth National Park, a newly established lowland forest that replaced a closed wooded grassland, and Mabira Central Forest Reserve (MCFR), located north of Lake Victoria, were also surveyed. Both surveyed forests represent moist tropical forest exhibiting characteristics similar to those of the Congo Basin forests (Mayaux et al. 2013). Rwenzori Mountain’s forest zone was dominated by *Podocarpus* species, with ferns and grasses filling the gaps naturally. Bwindi montane forest, displayed a mixture of dense herbs, shrubs, and vines, with natural clearings covered by ferns and grasses.

### Sampling of small mammals

Rodents and shrews were captured along transects of varying lengths where 30 snap traps and 15 Sherman live traps baited with peanut paste mixed with flour and silver fish were established. In Mabira, only Sherman traps were used to capture live rodents and shrews. The captured animals were brought to the camp and processed. Processing involved the collection of morphometric measurements and tissue samples (heart, liver, spleen, kidney, and blood). For live animals from Mabira, fresh blood was used for the preparation of thick and thin smears. Tissue samples (preferentially spleen) collected and stored in 96% ethanol were used for DNA barcoding to identify host species and detect trypanosomes. This yielded two independent data sets, one that could only be examined microscopically (blood smears), while the other could only be examined by PCR (tissue samples).

### DNA extraction, amplification, sequencing, and phylogeny

According to the manufacturer’s instructions, DNA was extracted from blood samples and tissue samples that had been preserved using RNAlater and 96% ethanol, respectively. DNA barcoding was used to identify species of mammalian hosts. The cytochrome b (cyt-b) gene was amplified and sequenced using a method outlined by Bryja et al. (2014). The sequences obtained were compared to unpublished data stored at the Institute of Vertebrate Biology, Czech Academy of Sciences, as well as datasets from recent taxonomic, phylogenetic, and phylogeographic studies focusing on specific mammalian genera. To amplify the 18S rRNA gene of trypanosomes, approximately 10 ng of previously extracted DNA was subjected to the trypanosomatid-specific nested PCR protocol described by Seward et al. (2017). The PCR products obtained were subjected to direct sequencing and the resulting sequences were then analyzed using Geneious software (version 10.0.6, https://www.geneious.com). To facilitate phylogenetic analysis, an alignment was generated using MAFFT v.7, incorporating (nearly) full-size 18S rRNA gene sequences obtained from GenBank. For phylogenetic reconstructions, we employed maximum likelihood (ML) using PhyML v.3.0.1 (Guindon et al. 2010) and Bayesian inference (BI) using MrBayes v.3.2.2 (Ronquist et al. 2012). Model optimization was performed using Model Test v.3.06 to determine the most appropriate model of sequence evolution. The general time-reversible substitution model with a mixed model for among-site rate variation (GTR + G + I) was identified as the best fitting model. Bootstrap analyses were conducted with 1000 replicates in ML. In the Bayesian inference analysis, we ran five million generations, sampling every 100 generations, while incorporating covarion. The default settings were maintained for all other parameters.

To assess whether there is a noteworthy disparity in the prevalence rate of trypanosomes based on various explanatory variables such as habitat and rodent sex, we utilized the Kruskal–Wallis equality nonparametric test. Furthermore, to investigate the factors that can be associated with the presence or absence of trypanosomes among different species of small mammals, we employed a binary logistic regression model. This model provides odds ratios as a measure of association.

## Results

The DNA sequences of the mammalian mt cyt-b gene precised the morphological identifications of the host species (Tables 1 and 2). A total of 711 small mammals belonging to 51 rodent and 12 shrew species were screened for trypanosomes, where 253 were microscopically tested using stained blood smears and 458 were screened by nested PCR.

**Table 1:** Results of the blood smear microscopy screening of rodents and shrews of the Mabira forest for trypanosome parasites

Rodent species	# Screened	# Positive	Prevalence %	Female	Male
*Aethomys hindei*	7			2	5
*Arvicanthis niloticus*	1			1	
*Crocidura* cf. *macmillani*	4			2	2
*Crocidura olivieri*	5			3	2
*Crocidura turba*	5			1	4
*Gerbilliscus validus*	8			3	5
*Hybomys univittatus*	1				1
*Hylomyscus stella*	70			29	41
*Lemniscomys striatus*	23	4	17	11	12
*Lophuromys stanleyi*	30			13	17
*Lophuromys ansorgei*	14			9	5
*Malacomys longipes*	1				1
*Mastomys erythroleucus*	9			6	3
*Mus bufo*	4	1	24	1	3
*Mus minutoides*	9			2	7
*Praomys jacksoni*	42	10	24	20	22
*Praomys mesonnei*	4			2	2
*Rattus rattus*	14	4	29	7	7
*Scutisorex congicus*	2			1	1

**Table 2:** Exploring trypanosome-small mammal interactions across diverse habitats of Uganda based on molecular data

				Habitat					Sex	
Rodent/shrew species	*Trypanosoma* genotypes	# Screened	# Positive	Bush fallow	Lowland forest	Grassland	Montane forest	Woodland	Female	Male
*Acomys cahirinus*		5	_	_	_	_	_	5	2	3
*Acomys percivali*	AF16, AF06a, AF06e	10	3	_	_	9	_	1	8	2
*Acomys wilsoni*	AF05i	6	1	_	_	2	_	4	2	4
*Aethomys hindei*	AF00	30	1	4	_	12	1	13	15	15
*Arvicanthis niloticus*		8	_	3	_	2	_	3	4	4
*Colomys goslingi*		1	_	_	_	_	1	_	1	_
*Cricetomys gambianus*		2	_	_	_	_	_	2	1	1
*Crocidura* sp. Kei	AF05p (2)	2	2	_	_	_	_	2	_	2
*Crocidura* cf. *fuscomurina*		1	_	1	_	_	_	_	1	_
*Crocidura hildegardeae*	AF08e (4)	8	4	2	_	2	4	_	4	4
*Crocidura lamottei-parvipes*	AF22d	7	1	_	_	6	_	1	4	3
*Crocidura littoralis*		2	_	2	_	_	_	_	1	1
*Crocidura monax*	AF08f (3)	3	3	1	_	_	2	_	2	1
*Crocidura montis-macmillani*		1	_	_	_	_	1	_	_	1
*Crocidura olivieri*	AF08e (2)	3	2	_	_	1	2	_	2	1
*Crocidura* sp. Wamba		1	_	_	_	_	_	1	_	1
*Crocidura turba*		2	_	_	_	1	1	_	2	_
*Dendromus* cf. *kivu*		1	_	_	_	_	1	_	1	_
*Deomys ferrugineus*		1	_	_	1	_	_	_	1	_
*Galegeeska rufescens*		1	_	_	_	1	_	_	_	1
*Gerbilliscus* cf. *bayeri*		3	_	_	_	_	_	3	2	1
*Gerbilliscus giffardi*		11	_	2	_	5	_	4	5	6
*Gerbilliscus* sp. 1		6	_	_	_	1	_	5	3	3
*Gerbilliscus* sp. 2		2	_	_	_	2	_	_	2	_
*Grammomys dryas*	AF01b (2)	5	2	4	_	_	1	_	3	2
*Grammomys macmillani*	AF01c	2	1	_	_	_	_	2	1	1
*Graphiurus murinus*	AF25	13	1	1	_	7	4	1	4	9
*Graphiurus vulcanicus*		3	_	_	_	_	3	_	1	2
*Hybomys lunaris*		8	_	1	_	_	7	_	6	2
*Hylomyscus denniae*	AF05l, AF05n	7	2	_	_	_	7	_	3	4
*Hylomyscus kerbispeterhansi*	AF05l	7	1	3	_	_	4	_	3	4
*Hylomyscus stella*		1	_	_	1	_	_	_	_	1
*Lemniscomys macculus*		6	_	_	_	3	_	3	2	4
*Lemniscomys striatus*	AF21a, AF08e	10	2	1	_	2	4	3	7	3
*Lophuromys ansorgei*		6	_	_	5	_	_	1	5	1
*Lophuromys dudui*		3	_	2	_	_	_	1	1	2
*Lophuromys stanleyi*	AF09c (4), AF05g	24	5	5	_	1	18	_	11	13
*Lophuromys woosnami*	AF24	12	1	_	_	_	12	_	7	5
*Mastomys erythroleucus*	AF22b, AF22c	30	2	2	2	15	1	10	19	11
*Mastomys natalensis*		22	_	1	_	17	1	3	12	10
*Mus bufo*	AF05o, AF05b (2), AF05m (3), AF05k (3)	28	9	5	_	4	10	9	18	10
*Mus* cf. *gratus*		6	_	4	_	_	_	2	1	5
*Mus* cf. *sorella*		3	_	_	_	1	_	2	2	1
*Mus minutoides*		4	_	_	_	3	_	1	3	1
*Mus musculoides*		9	_	2	_	3	_	4	4	5
*Mus sorella*		1	_	_	_	1	_	_	_	1
*Mus triton*	AF05b (2)	6	2	5	1	_	_	_	4	2
*Ochromyscus niveiventris*		38	_	_	_	21	3	14	21	17
*Oenomys hypoxanthus*	AF01d	8	1	3	_	_	5	_	5	3
*Otomys orestes/jacksoni*		3	_	_	_	_	3	_	2	1
*Otomys tropicalis elgonis*		1	_	1	_	_	_	_	1	_
*Praomys daltoni*		10	_	_	_	6	_	4	9	1
*Praomys degraaffi*		1	_	_	_	_	1	_	_	1
*Praomys jacksoni*	AF05a (5), AF05f (5)	49	10	8	1	_	31	9	27	22
*Praomys misonnei*		2	_	_	2	_	_	_	_	2
*Rattus rattus*	AF05b (2)	5	2	3	_	_	_	2	5	_
*Saccostomus mearnsi*		3	_	_	_	2	_	1	1	2
*Tachyoryctes splendens*		4	_	3	_	1	_	_	3	1
*Zelotomys hildegardeae*		1	_	_	_	_	_	1	1	_
Total		458	58	69	13	131	128	117	255	203

Microscopic screening of blood samples (S4) revealed a prevalence of 7%. Of 15 rodent and four shrew species, only four species were positive for trypanosomes (Table 1): *Rattus rattus* (29%), *Praomys jacksoni* (24%), *Mus bufo* (24%), and *Lemniscomys striatus* (17%). The proportion of positive individuals was marginally different between males (8%) and females (7%).

The nested PCR results revealed trypanosome infection in 17 rodents and five shrews out of 49 and 10 species, respectively, sampled across various habitat types in Uganda (Table 2) and indicated a total prevalence of 13%. There were no significant differences (Chi^2^ = 0.71, df = 1, *P* = 0.4) in prevalence between males (15.3%) and females (10.5%). Although the *R*^2^ value (0.045) from binary logistic regression odds ratios is low and does not explain a substantial portion of the variance in the data, it suggests that the odds of finding trypanosomes are slightly higher in males (OR = 1.14, *P* = 0.78) compared to females (OR = 0.96, *P* = 0.94).

The overall prevalence within the investigated mammalian genera ranged from 3.3% (*N* = 30) in *Aethomys* to 7% (*N* = 57) in *Mus*, 19% (*N* = 21) in *Acomys*, 20% (*N* = 15) in *Hylomyscus*, 40% (*N* = 5) in *Rattus*, 40% (*N* = 30) in *Crocidura*, and 43% (*N* = 7) in *Grammomys*.

The prevalence varied among the habitats studied, with montane forests showing the highest proportion of positive individuals (23.4%), followed by bush fallows (13.0%), woodlands (8.6%), grasslands (6.9%), and lowland forests (0%). It can also be inferred that the probability of finding positive trypanosome samples is lower in grasslands (OR = 0.50, *P* = 0.17) and woodlands (OR = 0.63, *P* = 0.36) compared to bush fallows (OR = 1.57, *P* = 0.13). On the contrary, it is more likely to find positive trypanosome samples in montane forests (OR = 1.76, *P* = 0.18) compared to bush fallows. Despite these variations, the prevalence among the sampled habitats did not show significant differences (Chi^2^ = 4.67, df = 4, *P* = 0.32).

**Fig. 2:**
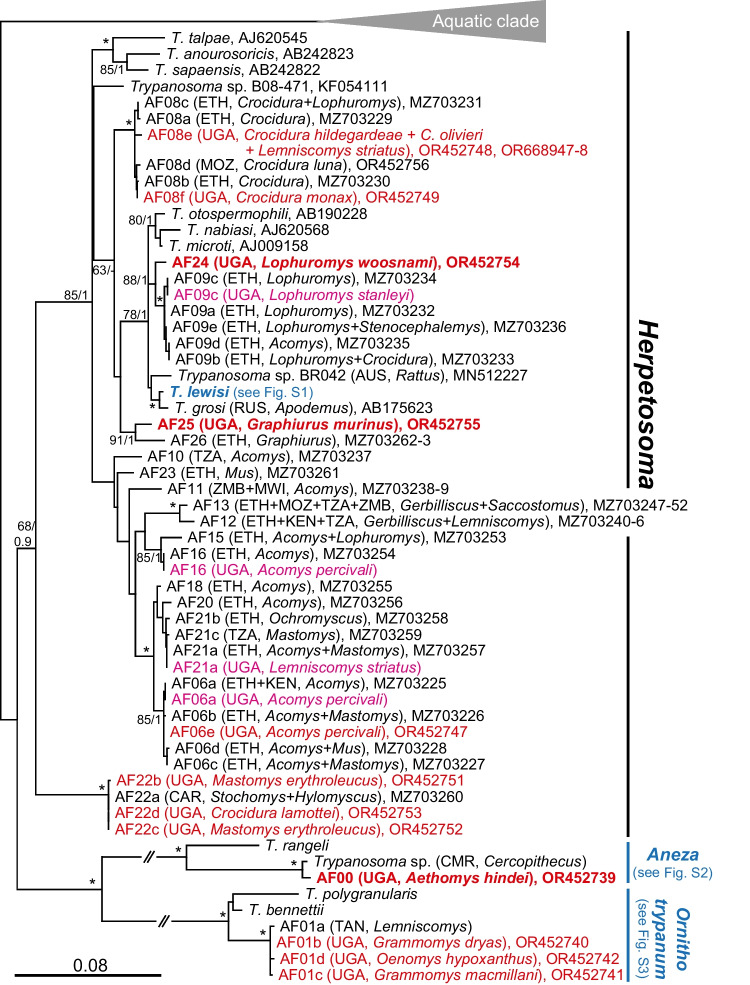
An 18S rRNA-based maximum likelihood phylogenetic reconstruction of all detected genotypes clustering into 11 phylogroups (putative species); already known genotypes are indicated by magenta, new genotypes are indicated by red (putative new species are in bold red); for details of the *Trypanosoma lewisi* subclade and the subgenera *Ornithotrypanum* and *Squmatrypanum*, see the individual subtrees (Supplementary figures S1, S2 and S3). Country ISO (International Organization of Standardization) alpha-3 codes and vertebrate host genera are indicated for all taxa/genotypes; asterisks mark branches with maximal statistical support (bootstrap values for maximum likelihood > 95, Bayesian posterior probabilities > 0.99); double-crossed branch is 50% of the original length; the scale bar denotes the number of substitutions per site

**Fig. 3:**
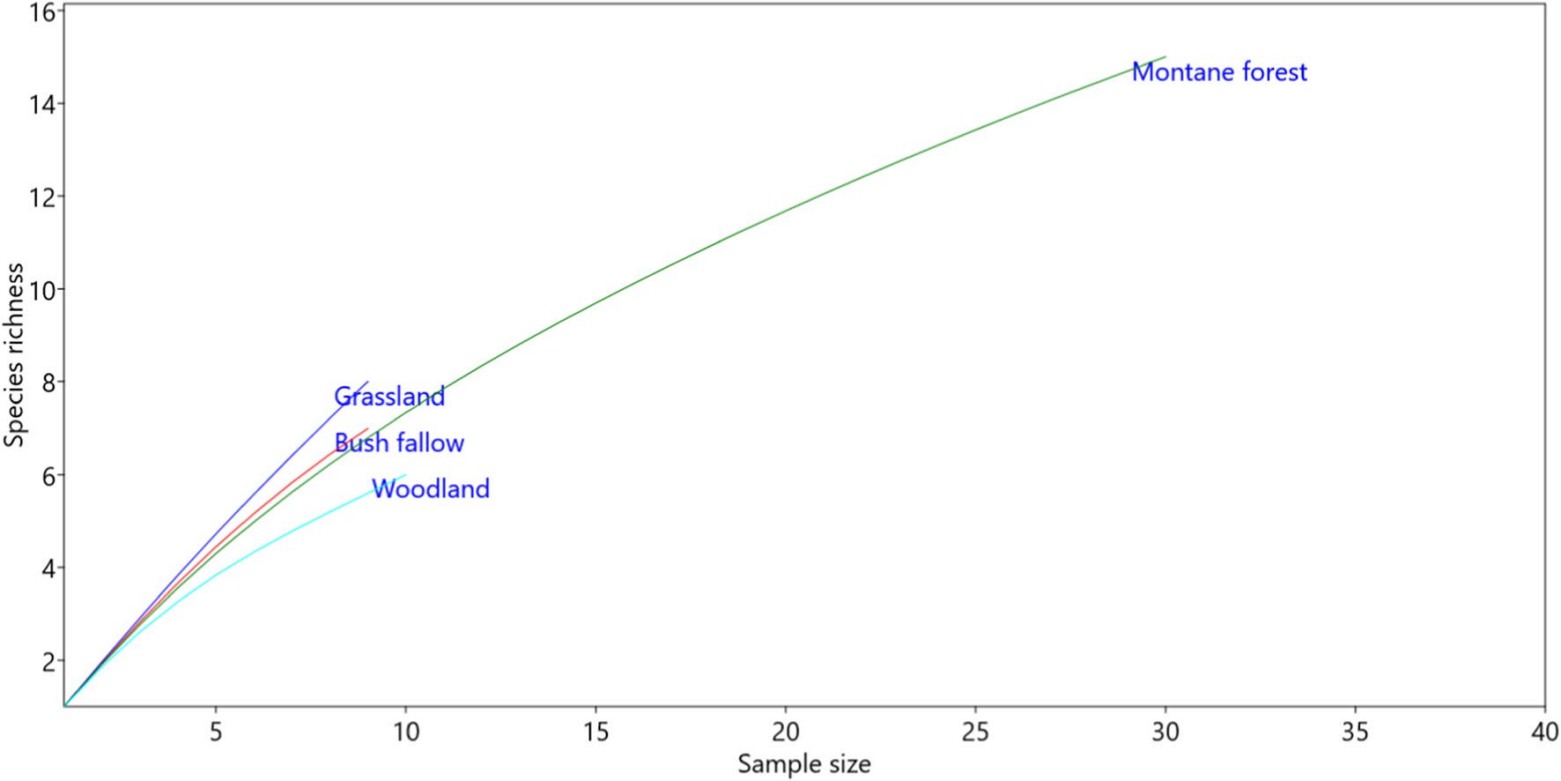
Species accumulation curves illustrating the *Trypanosoma* (*Herpetosoma*) species richness in different habitats. The curves depict the accumulation of species richness as the sampling effort increases

For *Herpetosoma* spp., we observed a positive correlation between the number of examined individuals per host genus and the number of detected trypanosome genotypes (weak correlation; *r* = 0.47) or trypanosome species (medium correlation; *r* = 0.6). The species accumulation curves for the *Herpetosoma* genotypes (Fig. 3) indicated that none of the curves in the surveyed habitats reached an asymptote.

**Fig. 4:**
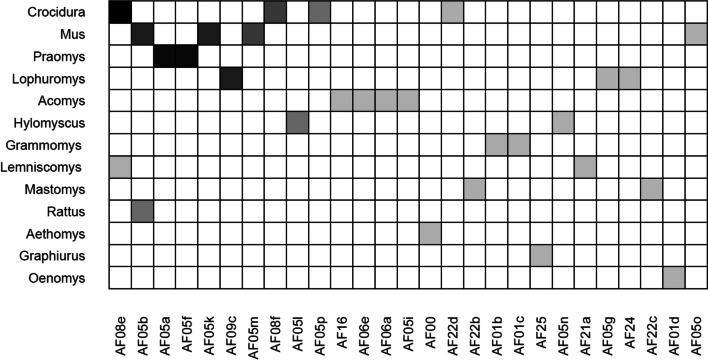
Host-trypanosome matrix plot illustrating the interactions between various host species and associated trypanosome genotypes detected in this study. The plot emphasizes the significant host specificity observed within most trypanosome phylogroups. The different shades in the cells of the web serve to highlight variations in the numbers of each genotype where lighter shades indicate lower values and darker shades represent higher values

Our analysis of 18S rRNA gene sequences revealed the presence of three trypanosome subgenera (Fig. 3). These include representatives of the *Herpetosoma* subgenus, as well as avian trypanosomes of the subgenus *Ornithotrypanum* (the *T. bennetti* clade) and mammalian trypanosomes of the subgenus *Aneza* (the *T. conorhini* clade). We identified 27 distinct genotypes (Fig. 3 and Table 2) grouped into 11 phylogroups (designated as AFxx after Votýpka et al. 2022), which exemplify different trypanosome species; five detected phylogroups were represented by more than one genotype (Figs. 2 and 4). Sequences of all detected genotypes/species are available under the GenBank accession numbers OR452739-56 and OR668939-48.

Most of the detected genotypes/species belong to the *Herpetosoma* subgenus (24 genotypes); among them, 12 genotypes and two putative species are new. For the species designated as AF08, which is clearly associated with insectivores, we detected two new genotypes and expanded the host range by three *Crocidura* species (*C. hildegardeae*, *C. monax*, and *C. olivieri*) and one rodent species, *Lemniscomys striatus* (Fig. 3, Table 2). Similarly, for the species AF22, which is considered basal (sister) of all other *Herpetosoma* species, we found three additional genotypes and expanded the host range by *Mastomys erythrolecus* and *Crocidura lemottei*. Eleven genotypes of the *T. lewisi* subclade are represented by five previously known (AF05a, AF05b, AF05f, AF05g, and AF05i) and six newly detected (AF05k-o) genotypes (for more details see S1). Within the other *Herpetosoma* species, we confirm the occurrence of previously detected genotypes (AF06a, AF09c, AF16, and AF21a) and add a new genotype AF06e. Newly designated species, represented by a single genotype each, were detected in *Lophuromys woosnami* (AF24) and *Graphiurus murinus* (AF25).

The subgenus *Aneza* is represented by a putative new species (AF00) with a single genotype (Fig. S2), while the recently established species AF01 (Votýpka et al. 2022) within the subgenus *Ornithotrypanum* is represented by three new genotypes (AF01b, AF01c, and AF01d; Fig. S3).

The phylogroup AF05 (Fig. S1), which represents the intricate *T. lewisi* subclade, was identified in all the habitats sampled, namely montane forest, low land forest, bush-fallow, grassland, and woodland. Eleven genotypes in our dataset are derived from nine rodent species belonging to seven genera: *Praomys* (with ten records representing two species), *Acomys* (1/1), *Crocidura* (2/1), *Hylomyscus* (3/2), *Lemniscomys* (1/1), *Mus* (9/4), and *Rattus* (2/1). While new genotypes AF05k, AF05o, and AF05m were detected exclusively in *Mus*, AF05l and AF05n were found exclusively in *Hylomyscus*, and AF05p was only detected in *Crocidura*.

While these eleven entire genotypes cluster within the *T. lewisi* subclade, only the genotype AF05b, identified in *Mus triton* from bush-fallow areas in Mt Elgon, *Mus bufo* from woodlands (Mt Kei) and montane forests (Mt Rwenzori), as well as commensal black rat (*Rattus rattus*) captured in mountain Rwenzori, belongs with 100% identity to the species *T. lewisi* sensu stricto.

The highest trypanosome genotypic diversity per host genus was in musk shrews (*Crocidura*) and mouse (*Mus*), both with five genotypes (representing 4 species), followed by the spiny mice (*Acomys*) with four genotypes (3 species) and brush-furred mice (*Lophuromys*) with three genotypes (3 species) (Fig. 4 and Table 2).

Although the detection of some trypanosome genotypes/species in various mammalian species/genera suggests relatively low host specificity (Fig. 4 and Table 2), a closer examination reveals a high level of host specificity in most trypanosome phylogroups/species. The AF05 phylogroup (the *T*. *lewisi* subclade) exhibited the lowest host specificity with the widest host range, as found in all habitats sampled and infected ten small mammalian species of seven genera (Fig. 4 and Table 2). However, even within the AF05 phylogroups, only one genotype, AF05b, was found in more than one host genera (Fig. 4). The remaining genotypes within this phylogroup demonstrated a high level of host specificity, infecting only one rodent / shrew genus (Fig. 4).

## Discussion

Our study showed that the rich variety of habitats in Uganda (Plumptre et al. 2019) contributes to the high diversity of captured small mammals, 53 species of 711 identified individuals. The prevalence of microscopy-determined trypanosomes is 7%, slightly higher than the 4% reported by Katakweba (2020) in Tanzania and the 3% documented by Shwan et al. (2016) in Mali. In contrast, the prevalence identified through nested PCR is 13%, which ranges with the findings of other studies such as Votýpka et al. (2022) with 11% in several sub-Saharan countries. This indicates that microscopy underestimates the true trypanosome prevalence, but also their diversity (Hoare 1972; Hamilton et al. 2009).

Similar infection rates, identified microscopically and by nested PCR, observed in both males and females, are consistent with previous findings (Wanyonyi et al. 2013; Votýpka et al. 2022). These results are based on the life cycle of the subgenus *Herpetosoma* transmitted by fleas, which do not exhibit significant preference between host sexes (Kiffner et al. 2013). The prevalence of trypanosomes did not differ significantly among the studied habitats, although the likelihood of encountering an infected small mammal was higher in the montane forests. This can be explained by the fact that fleas, which play a key role in *Herpetosoma* transmission, are known to have higher diversity and abundance in high elevations. For example, Baláž et al. (2020) highlighted that the majority of flea communities were found at higher elevations, and Mawanda et al. (2020) discovered fleas to be the most prevalent ectoparasites in small mammals in Bwindi (Uganda). Similarly, Meliyo et al. (2014) documented an increase in the abundance of small mammals and fleas with higher elevations in Lushoto (Tanzania). Our identification of a new *Herpetosoma* species in a specialized montane habitat rat emphasizes the scarcity of studies conducted in montane habitats in Africa and underscores the importance of studying the parasitofauna of endemic host species.

Among the 26 small mammalian genera examined for trypanosomes, 11 (42%) appear to be free of these parasites, as indicated in Table 2. In most genus-negative cases, only a limited number of individuals were screened, except the genus *Gerbilliscus*, where 22 individuals were tested. According to Votýpka et al. (2022), as long as a sufficient number of individuals have been examined, trypanosomes are likely to be found in almost every rodent genus, the only possible limitation being geographical location. Votýpka et al. (2022) documented that 87% of Tanzania *Gerbilliscus* host two closely related *Herpetosoma* species, AF12 and AF13, which were not detected in the present study. To better understand the geographical distribution of these two *Herpetosoma* species, more samples of *Gerbilliscus*, *Lemniscomys*, and *Saccostomus*, where AF12 and AF13 were previously detected, must be screened. The high prevalence of trypanosomes observed in the insectivore genus *Crocidura* can be attributed to its diet, as feeding on insects, including fleas, facilitating parasite transmission. Due to the colony lifestyle that facilitates flea transmission, the genus *Acomys* exhibits a high prevalence, which aligns with the findings of Votýpka et al. (2022), reporting similar prevalence and genotypes (e.g., AF06a and AF16).

### Diversity of *Herpetosoma* in Uganda’s small mammals

Within the subgenus *Herpetosoma*, we identified a significant number of genotypes representing at least 11 putative species, including two potentially new (AF24 in *Lophuromys* and AF25 in *Graphiurus*). Our findings align with Hoare’s concept (Hoare 1972) of high *Herpetosoma* host specificity.

Estimation of the true species richness, which encompasses both observed and undetected species, presents statistical challenges, especially in highly diverse assemblages with numerous rare species (Hortal et al. 2006; Chao and Chiu 2016). This estimation heavily relies on factors such as sampling effort, sample completeness, and, in the case of parasites, host specificity and the sensitivity of detection methods (Simo et al. 2011; Hamilton et al. 2009). Despite our screening of 63 small mammal species, the number of identified parasites remained relatively low, as indicated by the species accumulation curves. It is evident that a larger number of individuals per species need to be screened to reach an asymptote and also previous studies have demonstrated that increased sampling effort leads to the detection of a greater number of parasite species (Hoare 1972; Mafie et al. 2019; Votýpka et al. 2022).

Certain diverse small mammal genera, such as musk shrews (*Crocidura*), which boast the highest number of species among all mammal genera (Jenkins et al. 2009), have been found to harbor multiple trypanosome species. They are parasitized by at least four *Herpetosoma* species, including the *Crocidura*-specific phylogroup AF08, the *T. lewisi* subclade, and the basal/sister *Herpetosoma* phylogroup AF22. This study significantly extended the host of this basal clade by musk shrews (*Crocidura lemottei*) and multimammate rats (*Mastomys erythroleucus*), suggesting low host specificity and wide geographical distribution of this trypanosome species.

Votýpka et al. (2022) highlight the association of specific *Herpetosoma* species with endemic or geographically restricted hosts. A similar pattern is observed with species confined to montane forests, such as AF08f in *Crocidura monax* and the newly discovered *Herpetosoma* species AF24 in *Lophuromys woosnami*.

*Trypanosoma lewisi* and its spread in native small mammals.

Numerous countries around the world have reported the presence of *T. lewisi*, including South America (Lainson et al. 2004) and Southeast Asia (Ortiz et al. 2018), while in Africa, it has been documented in Nigeria, Niger, Mali (Dobigny et al. 2011; Schwan et al. 2016; Tatard et al. 2017), Uganda (Salzer et al. 2016), Egypt (Alsarraf et al. 2016; Dahesh and Mikhail 2016), Mozambique (Ortiz et al. 2018), and in Tanzania, Ethiopia, Kenya (Votýpka et al. 2022; where the *T. lewisi* subclade was represented by 11 different genotypes derived from 19 rodent species). Because the *T. lewisi* clade shows significant genetic variation across geographic distributions (Pumhom et al. 2015; Votýpka et al. 2022), coupled with its low host specificity (host jump), it presents a particular concern for potential spillover from invasive host species into native host species (Wyatt et al. 2008; Monique et al. 2020; Votýpka et al. 2022).

Among the 11 genotypes of the *T. lewisi* subclade identified in this study (S1), only the genotype AF05b shows 100% sequence similarity to the *T. lewisi* sensu stricto. Of the 59 native small mammal species examined, nine (*Crocidura* sp., *Hylomyscus denniae*, *Hylomyscus kerbispeterhansi*, *Acomys wilsoni*, *Lemniscomys striatus*, *Mus bufo*, *Mus triton*, and *Praomys jacksoni*) were found to be hosts of the *T. lewisi* subclade for the first time. Traditionally, the multimammate rat (*M. natalensis*) has long been considered the primary reservoir of *T. lewisi* s.s. in domestic and peridomestic environments, as documented in studies such as Maia da Silva et al. (2010) in Brazil; Ortiz et al. (2018) in South America, East Africa, and Southeast Asia; and Egan et al. (2020) in Australia. However, our survey yielded different results, since none of the 52 individuals examined (22 M*. natalensis* and 30 M*. erythroleucus*) was found to be infected by any genotypes of the *T. lewisi* (AF05) subclade. This finding supports the conclusion reached by Votýpka et al. (2022) that *Mastomys* does not play a significant role as a reservoir perpetuating the spread of *T. lewisi* s.s. in synanthropic and peridomestic rodents in East Africa.

Although AF05b, found exclusively in *Mus triton*, *M. bufo*, and *Rattus rattus* during this study, aligns perfectly with *T. lewisi* s.s., the classification of the remaining AF05 genotypes is not as clear-cut. This highlights the limitations of trypanosome taxonomy based solely on the 18S rRNA gene, as noted by Votýpka et al. (2022). According to their study, slight nucleotide variations (ranging from one to six substitutions) can make it challenging to determine whether newly detected genotypes simply contribute to the genetic diversity of *T. lewisi*, such as AF05h in Votýpka et al. (2022) or represent distinct species, such as AF05k.

### Other trypanosome subgenera

In addition to the *Herpetosoma* subgenus, which predominantly infects rodents, there are numerous other trypanosome species from various subgenera that have been identified in rodent populations (see Votýpka et al. 2022). Our analysis repeatedly detects an avian trypanosome (AF01) belonging to the subgenus *Ornithotrypanum* (the *T. bennetti* clade), which primarily infects birds, and a mammalian trypanosome (AF00) of the subgenus *Aneza* (the *T. conorhini* clade), parasitizing various mammalian species.

Within the mammalian subgenus *Aneza*, the AF00 sequence originated from Hinde’s rock rat (*Aethomys hindei*) captured in the grasslands of Ajai wildlife reserve, clusters with the sequences FM202493 of an unnamed trypanosome species found in the Greater white-nosed monkey (*Cercopithecus nictitans*) (Hamilton et al. 2009). This subgenus includes species such as *T. rangeli*, *T. conorhini*, *T. vespertilionis*, and *T. conorhini*; the last one has been documented in *Rattus rattus* (Hoare 1972; Mello 1979). Our finding is the first record of the subgenus *Aneza* in native African rodents.

Within the cosmopolitan subgenus *Ornithotrypanum*, infecting various bird species (Valkiūnas et al. 2011; Svobodová et al. 2017), four AF01 genotypes differing with only one to three nucleotides perfectly align with the sequence reported by Dobigny et al. (2011). Unlike other *Ornithotrypanum* species associated with birds, AF01 originates exclusively from rodents: *Praomys daltoni* from Niger (Dobigny et al. 2011); *Lemniscomys striatus* from Tanzania (Votýpka et al. 2022); and *Grammomys drays*, *Grammomys macmillani*, and *Oenomys hypoxanthus* from Uganda (this study, Fig. S3). These findings provide sufficient evidence that this trypanosome species regularly infects various sub-Saharan rodents.

## Conclusions

This study, documenting 27 genotypes of at least 11 different trypanosome species, has enhanced our understanding of the diversity, distribution, and prevalence of trypanosomes in small mammals in Uganda, while highlighting the limitations of microscopy screening and emphasizing the importance of PCR for accurate detection of parasite infections. We identified two new putative *Herpetosoma* species and demonstrated that some genotypes/species are associated with specific mammalian species or habitats. The detection of *T. lewisi* s.s. in two native *Mus* species expands the host range beyond the traditionally recognized reservoir, *M. natalensis*, emphasizing the need to consider a wider range of small mammal hosts in the epidemiology of *T. lewisi*. The findings underscore the need for continued research in diverse habitats and host species to fully elucidate the ecology and epidemiology of small mammal trypanosomes in Uganda.

### Supplementary Information

Below is the link to the electronic supplementary material.

Supplementary file1 (PDF 181 KB)

Supplementary file2 (PDF 364 KB)

Supplementary file3 (PDF 173 KB)

Supplementary file4 (PDF 217 KB)

## Data Availability

The datasets used in this study are available upon request from the corresponding author (waswasadic@gmail.com); however, data for all detected genotypes are available under the GenBank accession numbers OR452739-56 and OR668939-48.
