# Supplementary figures and images for: Trypanosome diversity in small mammals in Uganda and the spread of Trypanosoma lewisi to native species
Source: Parasitol Res. 2023 Dec 16;123(1):54. doi: 10.1007/s00436-023-08048-2 (PMC10724337; doi:10.1007/s00436-023-08048-2)

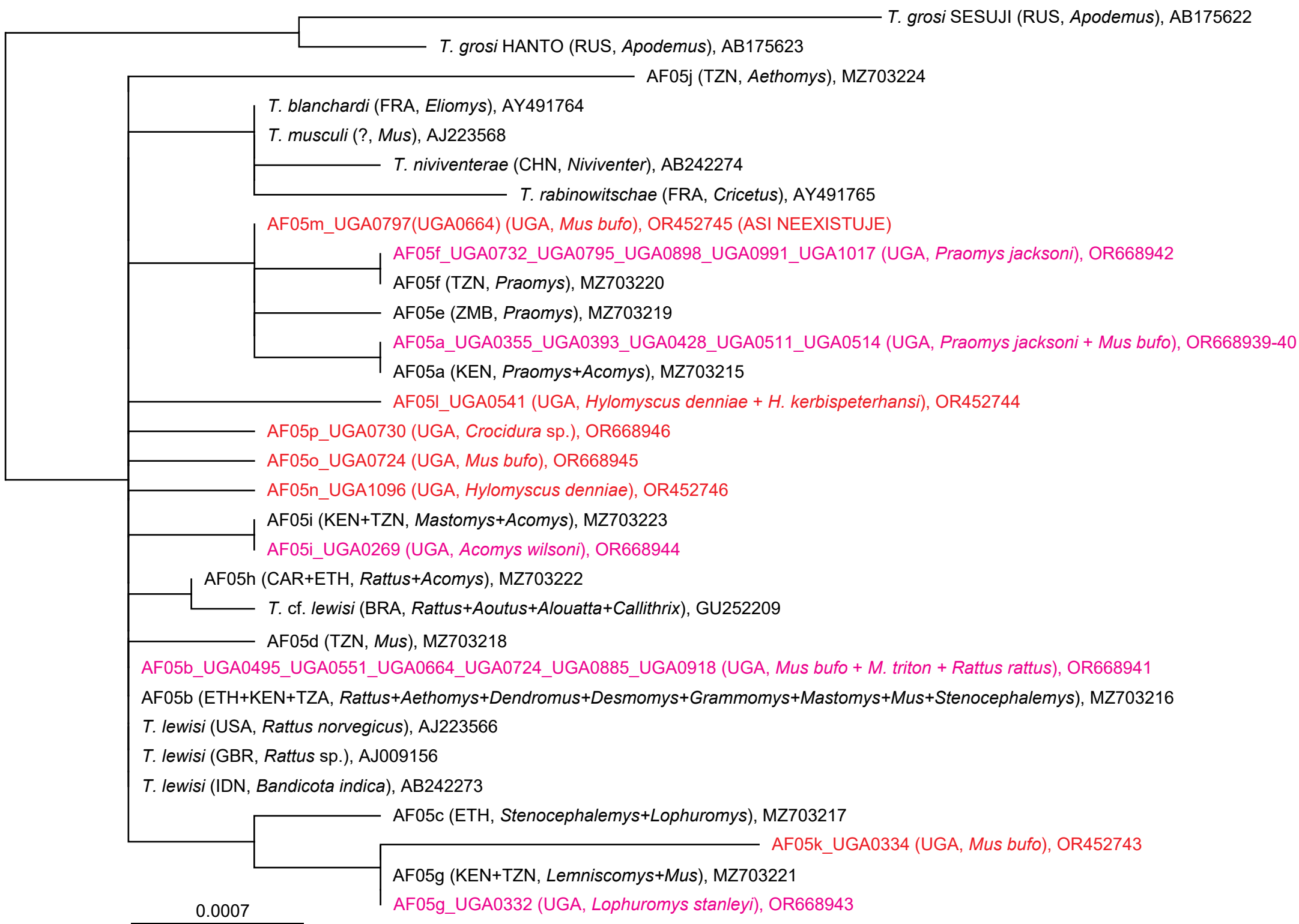

Supplement: Supplementary file 1 — Supplementary file1 (PDF 181 KB) [file 436_2023_8048_MOESM1_ESM.pdf]

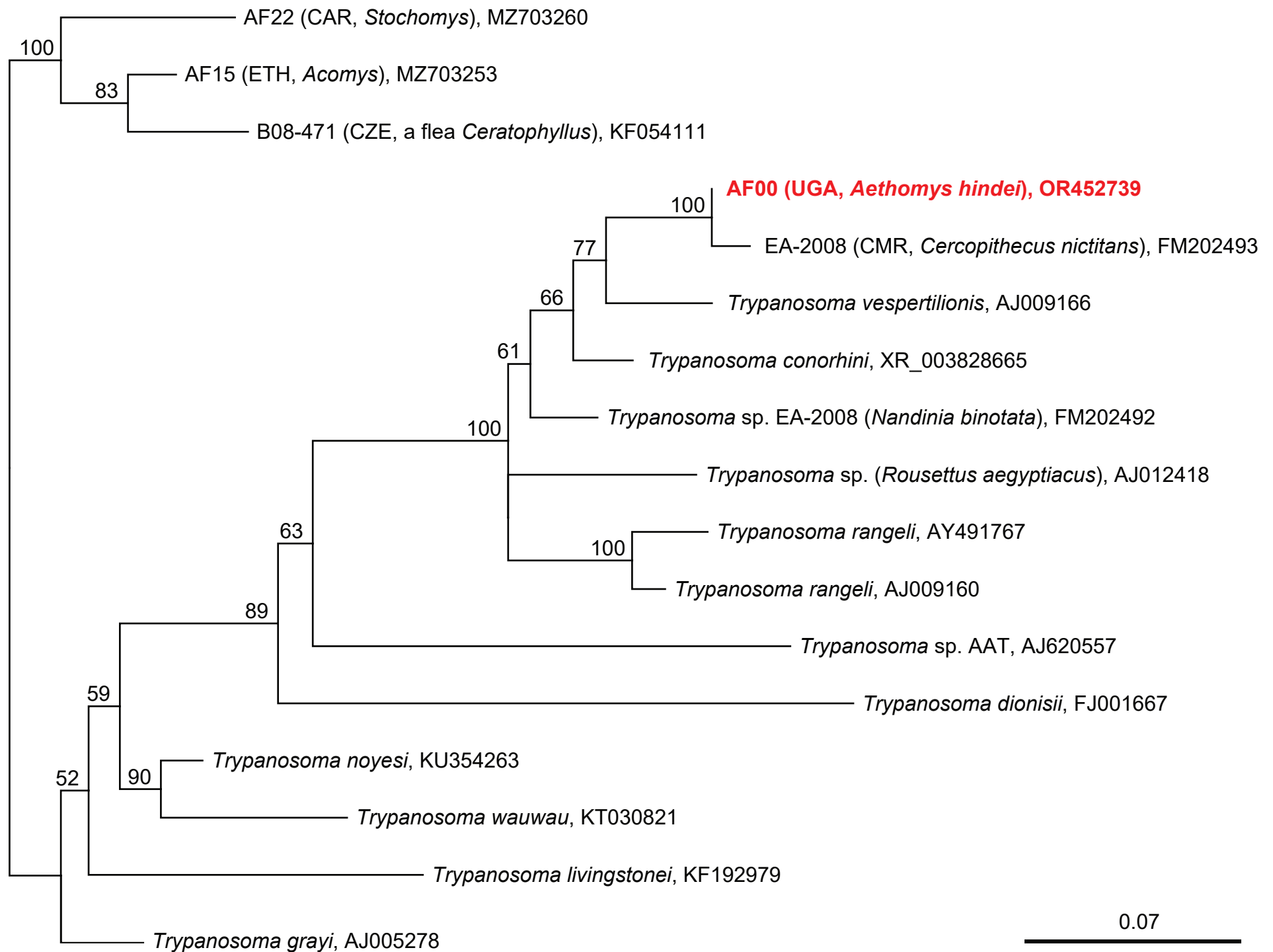

Supplement: Supplementary file 2 — Supplementary file2 (PDF 364 KB) [file 436_2023_8048_MOESM2_ESM.pdf]

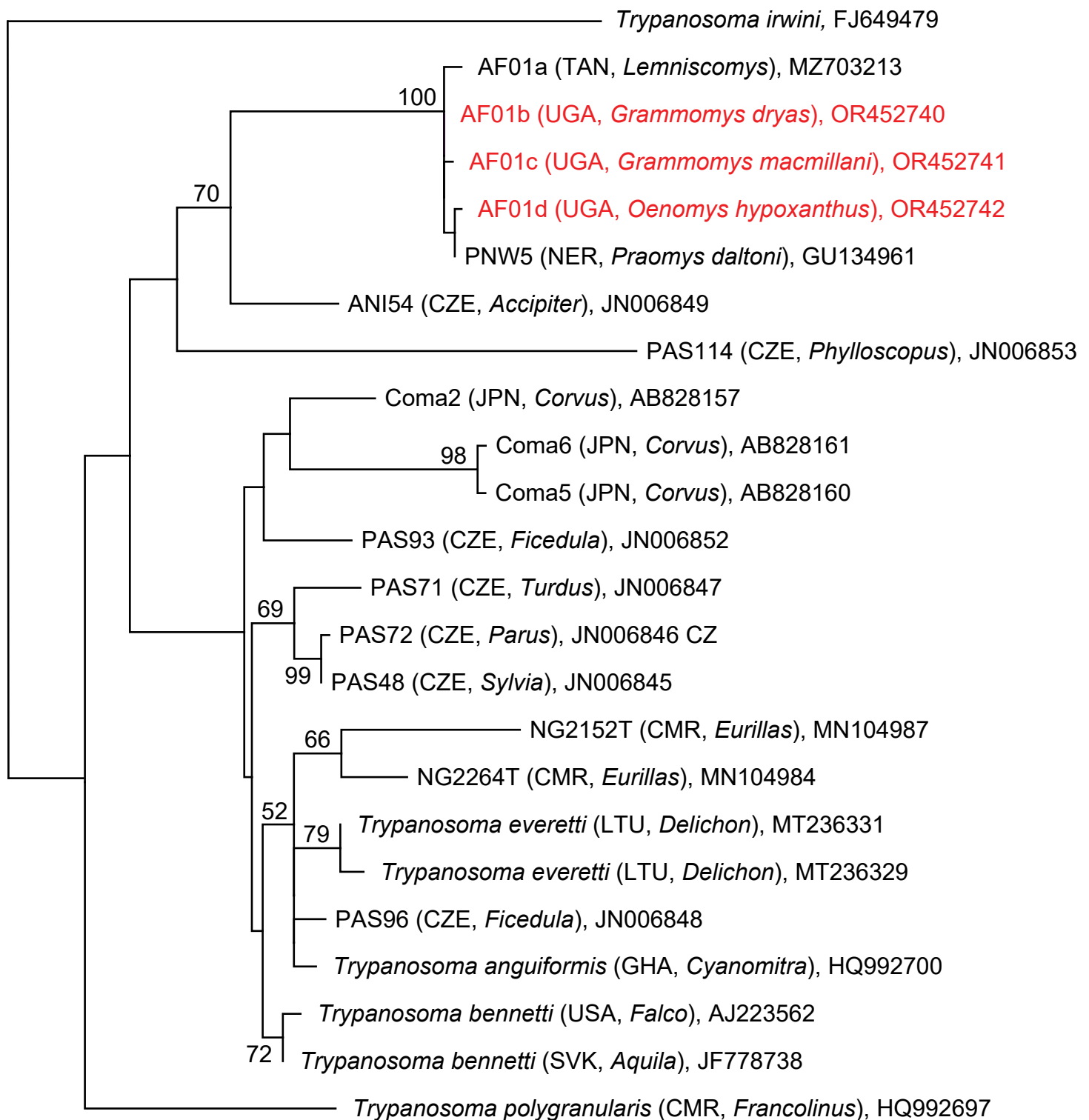

0.01

Supplement: Supplementary file 3 — Supplementary file3 (PDF 173 KB) [file 436_2023_8048_MOESM3_ESM.pdf]
