# Supplementary material for: Trypanosome diversity in small mammals in Uganda and the spread of Trypanosoma lewisi to native species
Source: Parasitol Res. 2023 Dec 16;123(1):54. doi: 10.1007/s00436-023-08048-2 (PMC10724337; doi:10.1007/s00436-023-08048-2)
Supplement: Supplementary file 4 — Supplementary file4 (PDF 217 KB) [file 436_2023_8048_MOESM4_ESM.pdf]

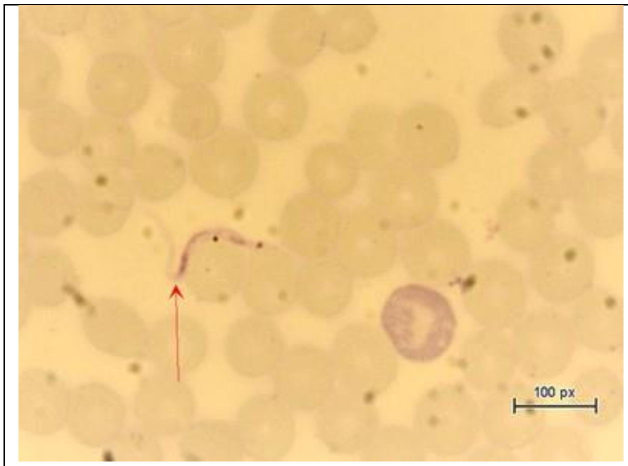

(a) *Rattus rattus*

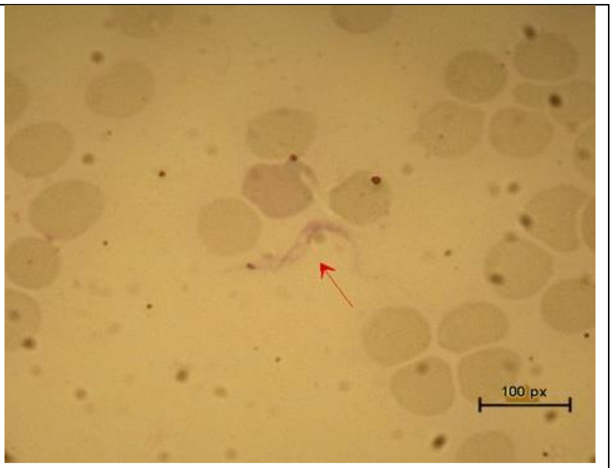

(b) *Lemniscomys striatus*

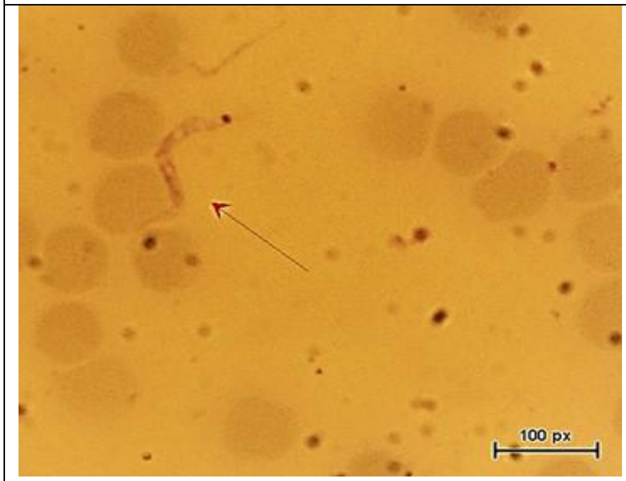

(c) *Mus bufo*

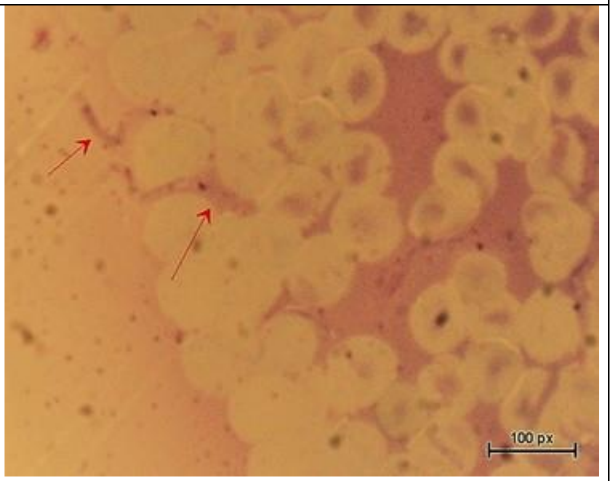

(d) *Praomys jacksoni*

Sample photographs of *Trypanosoma* (*Hepertosoma*) bloodstream form of rodent blood smear samples collected in Mabira
